# Influence of pairing in examiner leniency and stringency (‘hawk-dove effect’) in part II of the European Diploma of Anaesthesiology and Intensive Care: A cohort study

**DOI:** 10.1097/EJA.0000000000002052

**Published:** 2024-08-28

**Authors:** Stephen Sciberras, Markus Klimek, Bazil Ateleanu, Hugues Scipioni, Rodolphe Di Loreto, Joana Berger-Estilita

**Affiliations:** From the Department of Anaesthesia, ITU and Pain Management, Mater Dei Hospital, Msida, Malta (SS), Department of Anaesthesiology, Erasmus University Medical Centre, Rotterdam, the Netherlands (MK), European Society of Anaesthesiology and Intensive Care, Brussels, Belgium (MK, BA, HS, RDL, JBE), Department of Anaesthesia, University Hospital of Wales, Cardiff, UK (BA), Institute for Medical Education, University of Bern, Bern, Switzerland (JBE), CINTESIS@RISE - Centre for Health Technology and Services Research, Porto, Portugal (JBE) and Institute of Anaesthesiology and Intensive Care, Salemspital, Hirslanden Medical Group, Bern, Switzerland (JBE)

## Abstract

**BACKGROUND:**

The European Diploma of Anaesthesiology and Intensive Care (EDAIC) Part II examination is a supranational examination for anaesthesiologists.

**OBJECTIVE(S):**

We explore the impact of examiner pairing on leniency and stringency, commonly referred to as the ‘hawk-dove effect’. We investigate the potential variations in grading approaches, resulting from different examiner pairs and their implications for candidate performance.

**DESIGN:**

Retrospective cohort, observational design.

**SETTING:**

EDAIC Part II examination data from 2021 to 2023.

**PARTICIPANTS:**

Three hundred and twenty-five examiners across 122 EDAIC Part II single-day examination sessions.

**INTERVENTION(S):**

We analysed the influence of examiner leniency and examiner pairing on candidate performance in the EDAIC Part II using many-facet Rasch modelling.

**MAIN OUTCOME MEASURES:**

The study's main outcome measure was determining a leniency score among the examiner population. The study also aimed to assess how examiner pairing influenced candidate performance, as measured by their scores in the examination.

**RESULTS:**

During the study period, the number of examiners who participated in 2021, 2022 and 2023 were 253, 242 and 247, respectively. The median [IQR] single-day sessions attended were 7.0 [3 to 10]. The examination data revealed a mean leniency score of 0 (95% confidence interval (CI) −0.046 to 0.046), with the standard deviation being one-third that of the candidates’ ability scores. There were 1424 different pairs of examiners, with most pairs (97%) having only a one-point difference in marking. The mean leniency score for the pair of examiners was −0.053 (95% CI −0.069 to −0.037).

**CONCLUSION:**

The variations in grading approaches associated with different pairings emphasise the potential for the ‘hawk-dove effect’ to influence candidate performance and outcomes. Understanding these variations can guide curriculum development, examiner training and coupling, ensuring a balanced and equitable assessment process.

**TRIAL REGISTRATION:**

None

**KEY POINTS**

- The study examined 122 single-day examination sessions from 2021 to 2023, either conducted online, face to face, or hybrid in response to changing global circumstances.
- A total of 2905 candidates were included, and candidate participation increased each year.
- Analysis of 325 examiners highlighted a significant correlation between their leniency score and the number of sessions attended.
- A distinct difference in candidate performance was noted across exam formats, with hybrid sessions showing notably different scoring patterns compared with online and face-to-face sessions.

## Introduction

Examinations are integral components of medical education and play a pivotal role in assessing the clinical competence of aspiring healthcare professionals. These assessments provide a mechanism for evaluating a candidate's ability to apply theoretical knowledge in real-world clinical scenarios. However, the objectivity and consistency of such evaluations are susceptible to the influences of various factors, including examiner behaviour and inherent variations in stringency and leniency.^1,2^ The ‘hawk-dove effect’ refers to the observed variability in examiner leniency and stringency.^3^ Some examiners consistently demonstrate more stringent grading practices (‘hawks’), whereas others tend to be more lenient in their evaluations (‘doves’).^3^ This phenomenon has been identified in various medical assessments,^1–5^ reflecting the inherent subjectivity and complexity in evaluating clinical competence. The repercussions of such variations are multifaceted, affecting both candidates pass rates and the examination process's overall fairness, reliability and validity.^6^

The EDAIC Part II examination is a supranational examination designed to assess the competence of anaesthesiologists and illustrates how the ‘hawk-dove effect’ might manifest. This assessment is a multilingually conducted evaluation taken upon completion of training, divided into two sections, encompassing fundamental sciences and clinical subjects relevant to a specialised anaesthesiologist.^7^ An examiner's primary objective is to uniformly appraise a candidate's requisite knowledge at a consistently high level across European anaesthesiologists.^8–10^ A single-day oral examination session entails four distinct 25 min vivas for each candidate, including two focused on fundamental sciences and two on clinical topics. Each 25 minute viva entails 5 distinct subject questions with each question marked separately by each examiner. This comprehensive assessment evaluates a candidate's knowledge, clinical reasoning and decision-making skills in the dynamic and intricate anaesthesiology and intensive care fields.

Each candidate is evaluated by two examiners from different European countries culminating in interactions with eight examiners. The examination standard is set by the examiners.^11,12^ The decision to pass or fail the EDAIC exam is critical because it attributes a European competency title in Anaesthesiology and Intensive Care. Specialist training boards, employing institutions, governments, the community, and the candidates must be confident that the examination fulfils its purpose. Therefore, examiners need to correctly identify candidates who have acquired knowledge and competency that enables them to practice safely without direct supervision. The exam is frequently taken by younger candidates in their final year of residency or shortly after its completion, as it demonstrates their clinical competence and is a required or highly recommended component in several countries.^13^ This demographic context is crucial for understanding the stakes involved in the EDAIC exam and the importance of ensuring a fair and transparent assessment process.

Given the diverse and multifaceted nature of clinical scenarios encountered, the EDAIC Part II examination presents an ideal setting to investigate the impact of examiner leniency and stringency on candidate outcomes. Addressing the ‘hawk-dove effect’ within the EDAIC Part II examination context involves several intertwined factors, including examiner behaviour, candidate characteristics, test station complexity, and the inherent subjectivity of clinical judgment.

Examiner pairing, as a crucial component of the examination process, introduces an additional layer of complexity to the ‘hawk-dove effect’.^3^ The interaction between candidate and examiner(s), combined with the specific context of each assessment, can contribute to examiner stringency and leniency variations. The dynamics of these pairings may influence the overall assessment outcome, warranting rigorous investigation to elucidate the underlying mechanisms. This study seeks to elucidate the influence of examiner pairing on the ‘hawk-dove effect’ in the EDAIC Part II examination. We aim to investigate examiner leniency and stringency patterns and their interplay with candidate performance.

## Methods

### Ethics

This study followed the World Medical Association Declaration of Helsinki Ethical Principles for Medical Research Involving Human Subjects^14^ and complied with the Swiss Human Research Act. The Cantonal Ethics Committee of Bern (KEK Bern) Switzerland (Chairperson Prof. em. Dr med. Christian Seiler) waived ethical approval for this study (BASEC-number: Req-2024-00529) on the 12th April 2024. We followed the Strengthening the Reporting of Observational Studies in Epidemiology (STROBE) Statement.^15^

### Study design and setting

The study used a retrospective cohort analysis of data obtained from the European Diploma of Anaesthesiology and Intensive Care (EDAIC) Part II examination. This examination consists of *viva voce* questions designed to assess a candidate's knowledge and decision-making abilities in anaesthesiology. The setting for the study involved multiple examination centres where the EDAIC Part II examination is administered (https://esaic.org/professional-growth/edaic/part-ii-examination/#map).

### Study participants

The study participants can be categorised into two main groups:

#### Candidates

These are the individuals who took the exams during the 3-year period. These candidates were the primary subjects for the performance analysis, including their scores, and ability levels.

#### Examiners

These individuals were responsible for conducting and grading the exams. We examined various aspects of their performance, such as their leniency or stringency in marking, the number of sessions they attended, and how these factors correlated. The examiners’ behaviours and scoring patterns were essential for understanding the overall exam dynamics and assessing the fairness and consistency of the grading process.

Together, these two groups formed the core participants of the study, with their interactions and performances providing valuable data for the research analysis.

### Data collection and management

Data was collected for all EDAIC part II examinations from 2021 to 2023. After each examination, examiners scored their marks on a dedicated electronic platform, Ortrac (Orzone, Gothenburg, Sweden). We interrogated the Ortrac database and collected data on the questions from multiple EDAIC Part II exam administrations. Collected data included the number and distribution of exam sessions, including the format (online, face-to-face, hybrid), languages offered, and the geographic distribution of the sessions. In addition, we collected exam marking per question, candidate reference, examiner reference, type of paper, question difficulty, and examiner stringency/leniency. We also collected candidates’ performance data, the number of examiners involved and their participation over the years. The EDAIC Part II exam uses a three-point scoring system: 0 (failing response), 1 (borderline pass) and 2 (strong pass). The cumulative score from all examiners (4 vivas each with 5 questions, and 2 examiners, maximum score 80) is used to determine the final pass or fail outcome. Candidates must achieve a cumulative score of at least 60 points to pass the exam.

Stringency and leniency scores for each examiner were calculated using many-facet Rasch modelling. A stringent examiner (‘hawk’) consistently scores candidates lower than the average, resulting in a negative leniency score. Conversely, a lenient examiner (‘dove’) scores candidates higher than the average, resulting in a positive leniency score.

Candidate ability scores were determined using the Rasch model, which quantifies the overall performance of candidates across all examination components. Higher ability scores indicate better performance.

### Statistics

Descriptive statistics were employed to summarise the data, presenting categorical variables such as counts (*n*) and proportions (%) and medians with interquartile range [IQR]. Univariate analysis was performed with parametric tests, wherever appropriate. Data was first checked for normality and skewness using visual methods. Normality testing was done using the Shapiro–Wilk test. Whenever appropriate, *t* tests, Mann–Whitney *U* tests, Kruskal–Wallis's test and *χ*^2^ tests for contingency table analyses (two-sided) were used for univariate analysis. Most results did not show significant skewness, so mean values are reported. Significance was assumed where the *P* value was ≤ 0.05. Candidate ability scores and stringency and leniency scores for each examiner were calculated using many-facet Rasch modelling (MFRM). MFRM was performed using R package TAM v 4.1-4.^16^ Rasch modelling aims to identify the difficulty of questions, the ability of each candidate and also the leniency/stringency of the examiners.^17^ This is done by evaluating different responses grouped by the candidate and, in the case of MFRM, of the examiners. The results are presented as difficulty scores for topics, ability scores for each candidate and a leniency score for each examiner. Candidate's abilities are represented by the symbol θ. A θ score of zero indicates an average ability, with positive values representing candidates with above-average ability, and negative values representing those with below-average ability. It is important to note that this scale is non-linear, meaning that improvements in ability are not uniform across the range of values. The goodness of fit was assessed using Mean Square Errors (MSE), namely infit MSE and outfit MSE, both unstandardised and standardised. For the former, limits of 0.7 to 1.3 were considered acceptable, whereas a range of ±2 was used for the latter. Data was gathered into an Excel sheet (version 16.76, Microsoft, California, USA) and analysed using RStudio (RStudio: Integrated Development Environment for R, Version 2023.06.1+524, Boston, Massachusetts, USA), with R version 4.0.3 (The R Foundation for Statistical Computing, Vienna, Austria).

## Results

### Exam sessions

Between 2021 and 2023, a total of 122 single-day examination sessions were held in 15 centres in 9 countries. Although the COVID-19 pandemic in 2021 challenged previously established routines, 43 of the 122 sessions were performed during this period, all of which were conducted online.

### Candidates’ performance

The number of participating candidates in 2021 was 912; in 2022, it was 965; and in 2023, it was 1028. Over the three years, the mean number of candidates per single-day examination session was 23.8 (95% CI: 22.9 to 24.8), with a median score out of 10 of 7.37 [IQR: 7.1 to 7.7]. (Supplemental Digital File 1, Figure S1). The median score per viva given by examiners to candidates in 2021 was 7.6 [IQR: 7.2 to 7.8]; in 2022, it was 7.3 [IQR: 7 to 7.5], and in 2023, it was 7.3 [IQR: 7 to 7.5], *P* = 0.0049 (Supplemental Digital File 1, Figure S1).

Table 1 shows the total and frequency of each score. In 2022 and 2023, there were fewer scores of ‘2’ compared with 2021 (*P* = 0.0005). As a result of this, the mean pass rate was significantly higher (*P* = 0.0078) in 2021.

**Table 1:** Details of scores obtained by candidates, categorised by year

	2021	2022	2023	Total
Score	n = 912	n = 965	n = 1028	n = 2905
0	2222 (6.1)	2665 (7.0)	2493 (6.1)	7380 (6.4)
1	13 370 (37.0)	15 458 (40.3)	17 094 (41.8)	45 922 (39.2)
2	20 563 (56.9)	20 202 (52.7)	21 323 (52.1)	62 088 (54.4)
At least one ‘0’ assigned	536 (58.8)	602 (62.4)	602	1740 (59.9)
No ‘2’ assigned	4 (0.4)	4 (0.4)	10 (1.0)	18 (0.6)
Pass rate	57.4%	49.3%	49.7%	52.2%

Data are n (%)

Over the study period, 1740 out of 2905 (59.9%) candidates did not score a ‘0’ in any question. The maximum score in all sessions was 80 (maximum number of points possible). Twenty candidates (0.6%) achieved full marks. More candidates in 2021 achieved maximum points than in 2022 or 2023 [2021: 14 (1.5%) vs. 2022: 3 (0.3%) vs. 2023: 3 (0.3%)]. Similarly, the number of candidates who achieved a score of 60 or more was highest in 2021: 531 (58.2%) vs. 2022: 487 (50.5%) vs. 2023: 522 (50.8%), *P* = 0.0007.

### Rasch model

**Fig. 1:**
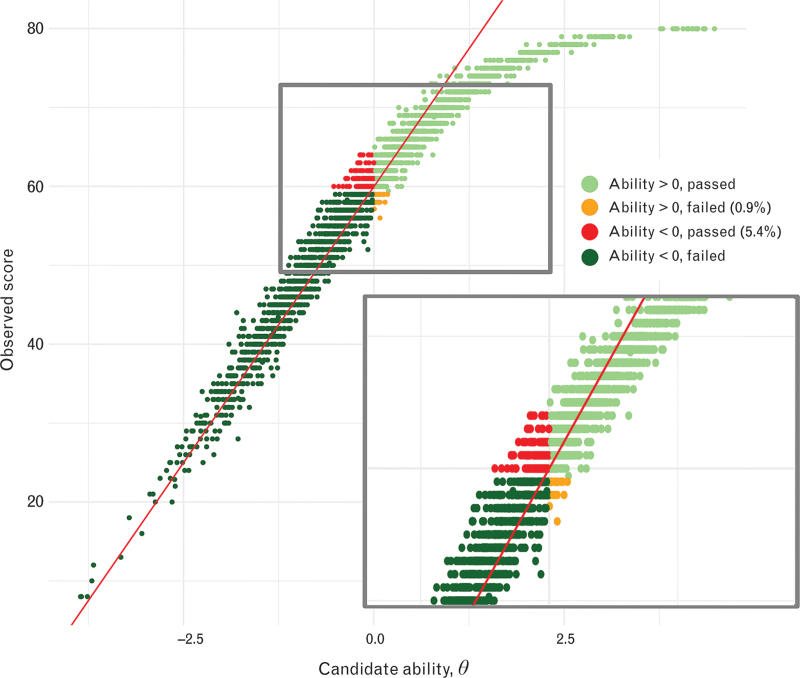
Comparison between observed mark and a candidate's ability score.

The ability scores of the candidates, as determined by the MFRM, are depicted in the Supplemental Digital File 2, Figure S2. The mean ability score was 0.004 (95% CI −0.037 to 0.046), with a standard deviation of 1.13. Candidate abilities decreased from 2021 to 2022 to 2023 (Supplemental Digital File 3, Figure S3). One-hundred and fifty-six (5.4%) candidates achieved a score of 60 or more despite having a candidate ability score less than 0, and 19 (0.6%) candidates achieved a score less than 60 despite having a higher candidate ability score. (Fig. 1).

The y-axis represents the total observed score for each candidate across all questions. The x-axis represents the candidate's ability score (θ), where zero indicates average ability, positive values indicate higher-than-average ability, and negative values indicate lower-than-average ability. Ability scores are calculated using the Rasch Model, and the scale is non-linear. The inset highlights the range of observed scores near the passing threshold of 60.

### Examiners’ performance

The cohort had 325 different examiners involved in the 122 single-day sessions. In 2021, there were 253 examiners, 242 in 2022, and 247 in 2023. The median number of single-day sessions attended by the examiners over these 3 years was 7.0 [IQR: 3 to 10], range from 1 to 61. The mean leniency score was 0 (95% CI: −0.046 to 0.046). The standard deviation across all examiners was 0.42. This is considerably less than the standard deviation of the candidates’ abilities, which was 1.13. This would indicate that the candidate's performance is responsible for around three times more variance in the marks than the examiners’ performance. Furthermore, the spread of the leniency scores follows a normal distribution (Supplemental Digital File 4, Figure S4), indicating that it is difficult to separate the examiners into two groups of hawks or doves.

**Fig. 2:**
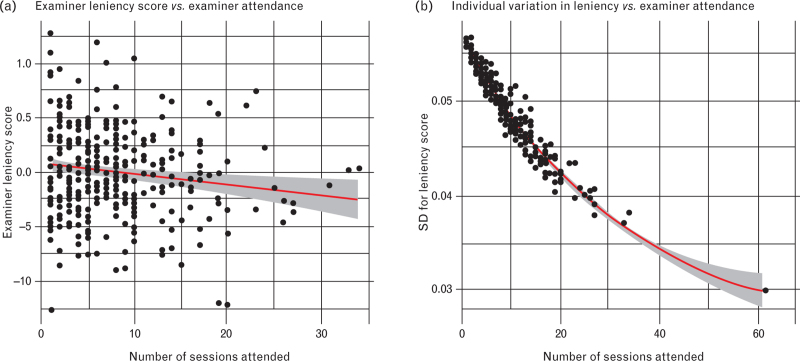
Examiner leniency score compared with the number of sessions attended by each examiner during 2021 to 2023.

There was a statistically significant correlation between the leniency score and the number of sessions attended by an examiner (linear regression: leniency-score ∼ *n* – estimate: −0.009, *P* = 0.009, model = 0.009), which persisted even when examiners who attended only one session were excluded from the analysis (Fig. 2). However, this effect was small (*R*^*2*^ = 0.018). The variance in the leniency score for each examiner seems to decrease with an increase in the number of examination sessions attended.

The *x* axis represents the number of sessions attended, and the *y* axis shows the leniency score. The individual variation, indicated by the standard deviation of the mean leniency score, is also plotted against the number of sessions attended.

### Comparison of markings

**Fig. 3:**
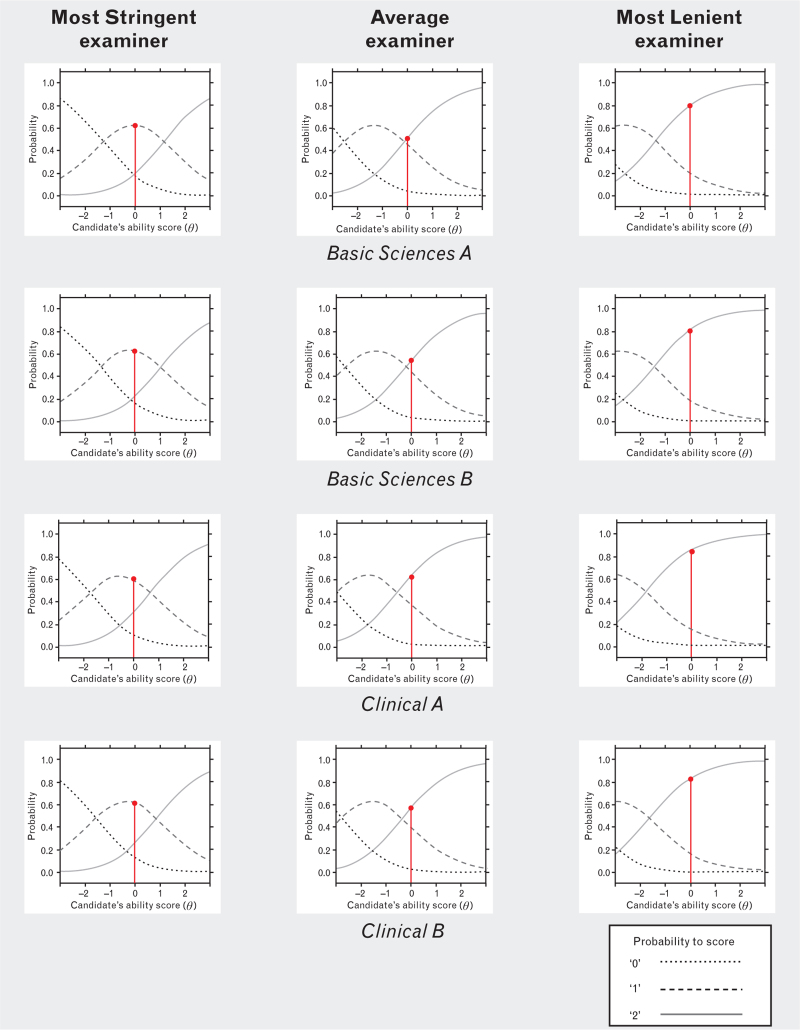
Probability scores of three actual examiners during the period investigated, showing the probability of scoring a candidate based on their ability (*θ*).

Figure 3 shows a comparison of probabilities of allocating a particular score, depending on the candidate's ability. For this comparison, we chose the most stringent examiner, the most lenient examiner and the examiner with the leniency score closest to zero.

Probability scores for three actual examiners during the period investigated, showing the probability of scoring a candidate based on their ability (*θ*). This figure demonstrates the impact of examiner leniency and stringency on the scoring probabilities, highlighting the variability in examiner behaviour. The dotted line shows the probability of scoring ‘0’, the hashed line the probability of scoring ‘1’, and the solid line shows the probability of scoring ‘2’. This is shown for Basic Sciences vivas A and B, and the Clinical vivas A and B. It should be noted that for a candidate ability (*θ*) of ‘0’, then the most stringent examiner (left column) would most likely score a ‘1’ in each section (vertical red line intersects the hashed line), whereas an average (middle column) and the most lenient examiner (right column) would mark ‘2’ in each section (vertical red line intersects the solid line).

### Pairs of examiners

**Fig. 4:**
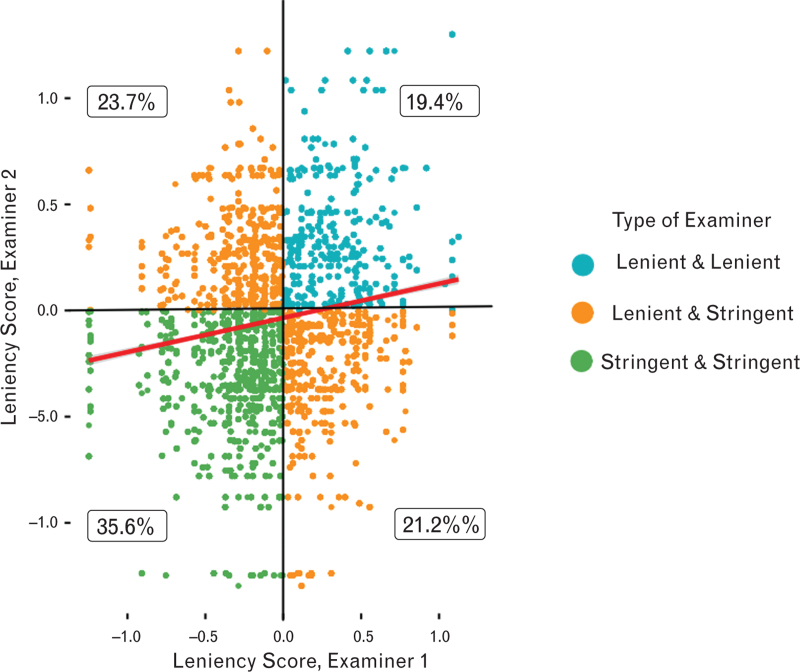
Scatterplot of examiner leniency scores between pairs of examiners.

In total, there were 1424 different pairs of examiners. Most pairs scored, in median, 1 point difference [IQR: 0 to 1], with a difference of scoring greater than 2 points occurring in 368 instances (3.2%). The combination of a stringent (negative leniency score, ‘hawk’) and a lenient (positive leniency score, ‘dove’) examiner was most common, at 44.9%, followed by a pair of stringent (hawk) examiners (35.6%) and finally by a pair of lenient (Dove) examiners (19.4%) (Figure 4). The leniency scores were averaged for each pair of examiners: the mean leniency score for the pair of examiners was −0.053 (95% CI: −0.069 to −0.037). The average difference between the leniency scores of each examiner in a pair was 0.42 (95% CI: 0.40 to 0.42), with a maximum difference of 1.91.

The *x*-axis represents the leniency score of the first examiner, and the *y*-axis represents the leniency score of the second examiner. The plot illustrates the distribution of leniency scores for pairs of examiners. Note that although four combinations are possible, two of such pairs would be equivalent. The red line shows the regression for the leniency scores of the two examiners – this is nearly horizontal, indicating little correlation between the leniency scores of the two examiners.

### Online vs. face-to-face performance

**Fig. 5:**
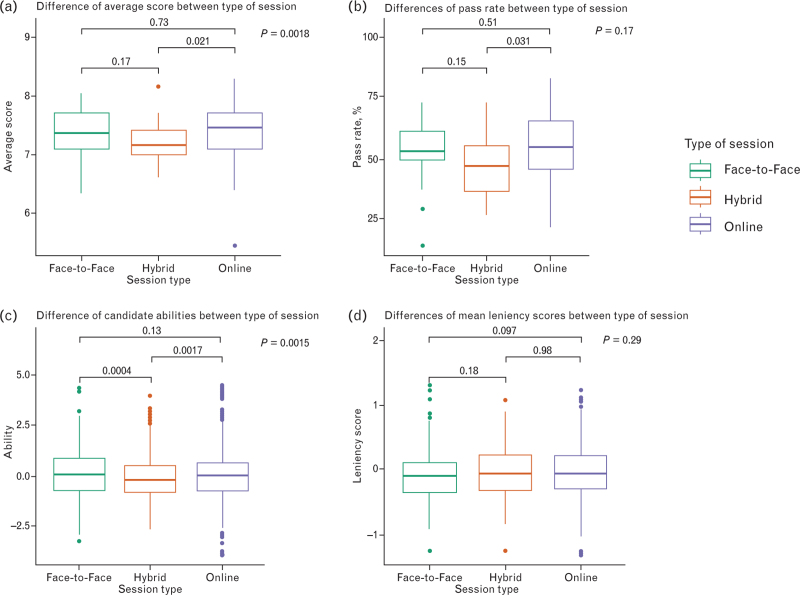
Comparison of various outcomes between Face-to-Face, Hybrid and Online sessions.

Figure 5a compares the average score between each type of exam session. This was statistically significant (*P* = 0.018), with the main significance being between the hybrid and online sessions. Despite this, the pass rate between the different types of sessions was not statistically significant (*P* = 0.17), Fig. 5b.

(a) Comparison of average score obtained by candidates. (b) Comparison of pass rates between types of sessions. (c) Comparison of mean candidate abilities. (d) Comparison of mean leniency scores of examiners. The *x*-axis in each subplot represents the type of session, and the *y*-axis shows the corresponding outcome measure.

The candidates’ abilities were statistically significantly different (*P* = 0.0015) between types of sessions (Fig. 5c). The pairwise comparison shows no difference between online and face-to-face sessions, with differences only for hybrid sessions. There was no relationship between the mean leniency scores per session and the type of session (Fig. 5d).

### Goodness of fit

The model was able to explain 88.7% of the variability in exam outcomes, which means that the factors included in the model (candidate abilities, examiner performance and the type of viva) were significant in understanding why different candidates received different scores. The fit statistics for the examiners showed acceptable unstandardised infit and outfit MSE values but not standardised values. The ‘underfit’ suggests that the examiners’ scores were not as finely differentiated as might be desired, which could mean that the examiners were not distinguishing as sharply between different levels of candidate performance as the model expected. For a more complex description of the results of the Goodness of Fit model, refer to Supplemental Digital File 4.

## Discussion

For the first time, we employed Rasch modelling to analyse the EDAIC Part II, aiming to ensure fairness as examination centres and candidate numbers grow. The hawk-dove effect, noted by Osler since 1913,^18^ underscores the significant influence of examiners on results. Although strict examiners may impact outcomes, our analysis aims to guide a strategy moving forward rather than evaluate individual examiners.

The average pass rate of 52.2% is comparable with other major medical exams.^19–21^ The pass rate spiked in 2021, likely because of COVID-19's impact on the format. With the introduction of new formats and many new questions in 2022, the pass rate stabilised at just under 50%. The pass mark aligns with the mean candidate ability score, with a linear relationship, except for higher ability candidates. Notably, only a small percentage of candidates achieved high or low scores despite their ability levels, possibly because of chance.

There are few documented examples of similar analyses for other examinations and residency programs.^2,3,22^ McManus *et al.*^3^ conducted a comparable evaluation of the MRCP (UK) PACES examination, revealing that individual examiner traits influence candidate marking. This was also described in other studies.^23^ Indeed, examiner variance in various exams accounts for 12 to 45% of the total variance.^1,3^ Although our investigation showed examiners contribute around 26% of the total variance, candidate performance remains the primary determinant of marking.

Across 122 sessions and 325 examiners over 3 years, each examiner encountered an average of 84 candidates. This mirrors findings from McManus *et al.*^3^ The average leniency score of zero suggests no overall bias among examiners regarding candidate performance. Moreover, the leniency score's normal distribution indicates no clear outliers identifiable as either doves or hawks, aligning with patterns seen in other examinations.^1–3^

Although we did not compare leniency scores across examiner characteristics like gender or age, we did examine the association between leniency scores and the number of sessions attended by each examiner. We found a dual effect: as examiners attended more sessions, they adopted a stricter approach, leading to less variability in their scoring. This trend, also observed in other studies,^3^ may also stem from increased examiner confidence in their judgments with more experience or exposure to diverse candidate performances.

In the EDAIC, each candidate was evaluated by a pair of examiners who scored them independently in the assessment process. These pairs are usually chosen randomly, but when a new examiner is involved, they are paired with a more experienced colleague for guidance. The combined leniency score of these latter pairs was nearly zero, indicating that the pairing system effectively countered bias and minimised the impact of individual examiner tendencies, whether lenient or strict. The largest difference in leniency score observed was 1.9 between two senior examiners, where one was significantly more lenient than the other. Yet, even in this case, the pair's average leniency score remained relatively balanced at −0.29. This suggests that the current process of pairing examiners effectively neutralises any bias or tendencies towards leniency or strictness that individual examiners may have. Even when there is a notable difference in leniency between two examiners in a pair, the combined leniency score tends to remain balanced.

Since the onset of the COVID-19 pandemic, several postgraduate examinations have been redesigned to accommodate an online format, necessitating corresponding adjustments and requirements.^24^ Although some examinations have transitioned entirely to online formats, the EDAIC Part II offers face-to-face, online and hybrid options. In hybrid sessions, examiners convene at a designated centre, akin to face-to-face sessions, while candidates undertake the examination remotely. Including both online and face-to-face formats in 2022 and 2023 underscores adaptability and flexibility in response to evolving circumstances. This commitment reflects a dedication to offering candidates choices in examination formats, ensuring accessibility and continuity across diverse conditions. Encouragingly, the pass rates across the three examination types showed no significant differences. Additionally, the type of examination did not affect examiner leniency scores, indicating examiners’ adeptness at transitioning between different examination modalities. However, candidate abilities appeared to vary across different session types. Yet, this discrepancy should be interpreted cautiously. From a candidate's standpoint, a hybrid session resembles an online one. Therefore, any observed differences in abilities between online and hybrid sessions are probably attributed to other factors.

The adaptability of hybrid examination formats, as demonstrated in our study, suggests that combining online and face-to-face assessments can maintain examination integrity while providing flexibility. Other disciplines can consider this model to accommodate diverse candidate needs and unforeseen circumstances, such as global pandemics. Our findings also underscore the importance of rigorous examiner training and calibration to minimise variability in grading. The study highlights the effectiveness of pairing examiners with different experience levels and leniency. This practice is key for the EDAIC Part II examination and other medical and professional examinations where subjective judgment plays a significant role, balancing examiner biases and ensuring more consistent and fair candidate evaluations. In addition, implementing many-facet Rasch modelling (MFRM) or similar statistical techniques can help identify and correct for examiner leniency and stringency. This approach can be adopted by other examination bodies to enhance the accuracy and fairness of their assessment processes.

The goodness-of-fit statistics revealed challenges in differentiating examiner scores, indicating that examiners may not accurately distinguish candidate performance levels. This is a critical issue for the ESAIC to consider, as it impacts the accuracy and reliability of the assessment process. Our findings suggest that new examiners tend to be less strict in scoring, which may contribute to the observed variability in examiner behaviour. To address this limitation, we recommend the following measures: develop comprehensive training programs for new examiners that emphasise scoring judgment and provide guidelines for distinguishing between different levels of candidate performance; implement regular feedback and monitoring systems to assess examiner performance and provide ongoing support. This could help identify areas where examiners may need additional training or guidance. By implementing these recommendations, the ESAIC can improve the accuracy and reliability of the EDAIC Part II examination, ensuring a fair and balanced assessment process for all candidates.

Although our study offers valuable insights into the dynamics of the EDAIC Part II examination and the impact of examiner pairing and session types on candidate performance, it is important to recognise its limitations. The findings are based on data from the EDAIC Part II exam and may not directly apply to other medical exams or specialties. Unique factors like format, content and candidate demographics of the EDAIC exam might restrict generalisability. We relied on retrospective analysis of data from the exam's electronic platform. This approach may introduce biases or inaccuracies, like missing records, which could affect the validity of our findings. Although we explored examiner leniency and stringency patterns, we did not delve into individual examiner traits like gender or clinical experience. These factors could influence examiner behaviour and warrant further investigation. Our focus was primarily on examiner performance, overlooking candidate traits beyond performance scores. Training, experience and cultural background could also impact performance and should be considered in future research. The observational nature of our study limits our ability to establish causal relationships between variables. Although we identified correlations, establishing causality would require experimental manipulation or longitudinal studies. There may be a bias, particularly in self-reported data from examiners. Factors like social desirability bias or subjective interpretations of scoring criteria could influence results. Our focus was primarily on candidate performance scores and examiner leniency, with limited consideration of other outcomes such as candidate satisfaction or long-term career impacts. Additionally, our Rasch modelling analysis assumed unidimensionality, meaning that a candidate's ability remains consistent across all questions. However, individual candidates may perform better in certain subjects regardless of examiner leniency, as indicated by deviations in ability scores, particularly among candidates scoring close to zero. Finally, although our study provides a comprehensive analysis of examiner leniency and stringency using multifacet Rasch modelling, we acknowledge that it does not explore individual examiner characteristics such as gender, age or clinical experience. These factors could provide a more nuanced understanding of the ‘hawk-dove effect’ and are important areas for future research. That being said, previous studies suggest no significant differences based on gender or age.^1,3^ Investigating these characteristics could help develop targeted interventions for examiner training, further enhancing the fairness and reliability of the EDAIC Part II examination. Future studies should consider incorporating these variables better to understand their impact on examiner behaviour and candidate outcomes. We also acknowledge that this exam's unique format, content and candidate demographics may limit the broader applicability of our results. The EDAIC Part II is distinct in its supranational scope, multilingual administration and specific focus on anaesthesiology and intensive care, which may not be directly comparable to other medical examinations or specialities. The findings presented here should be interpreted cautiously when considering other contexts.

However, this study has the potential to pave the way for evidence-based strategies to enhance the objectivity and fairness of the EDAIC Part II examination. The insights from this research can inform the development of targeted examiner training programs, standardised assessment protocols and quality assurance measures. By quantifying the impact of the ‘hawk-dove effect’ and uncovering its underlying factors, we can cultivate a more equitable and dependable examination process. This process will accurately mirror a candidate's clinical competence in anaesthesiology and intensive care, ensuring more rigorous and transparent evaluation.

In conclusion, exploring examiner leniency and stringency, particularly within the framework of examiner pairing, holds significant implications for the validity and fairness of the EDAIC Part II examination. This study represents a step towards unravelling the intricate dynamics of the ‘hawk-dove effect’ and its potential influence on candidate outcomes. Through a rigorous scientific approach, we aim to advance medical education assessment practices and ensure the integrity and accuracy of evaluating clinical competence in anaesthesiology and intensive care by the EDAIC Part II.

## Supplementary Material

Supplemental Digital Content

## Supplementary Material

Supplemental Digital Content

## Supplementary Material

Supplemental Digital Content

## Supplementary Material

Supplemental Digital Content

## Supplementary Material

Supplemental Digital Content
