# Supplementary figures and images for: Influence of pairing in examiner leniency and stringency (‘hawk-dove effect’) in part II of the European Diploma of Anaesthesiology and Intensive Care: A cohort study
Source: Eur J Anaesthesiol. 2024 Aug 28;41(12):921–31. doi: 10.1097/EJA.0000000000002052 (PMC11556864; doi:10.1097/EJA.0000000000002052)

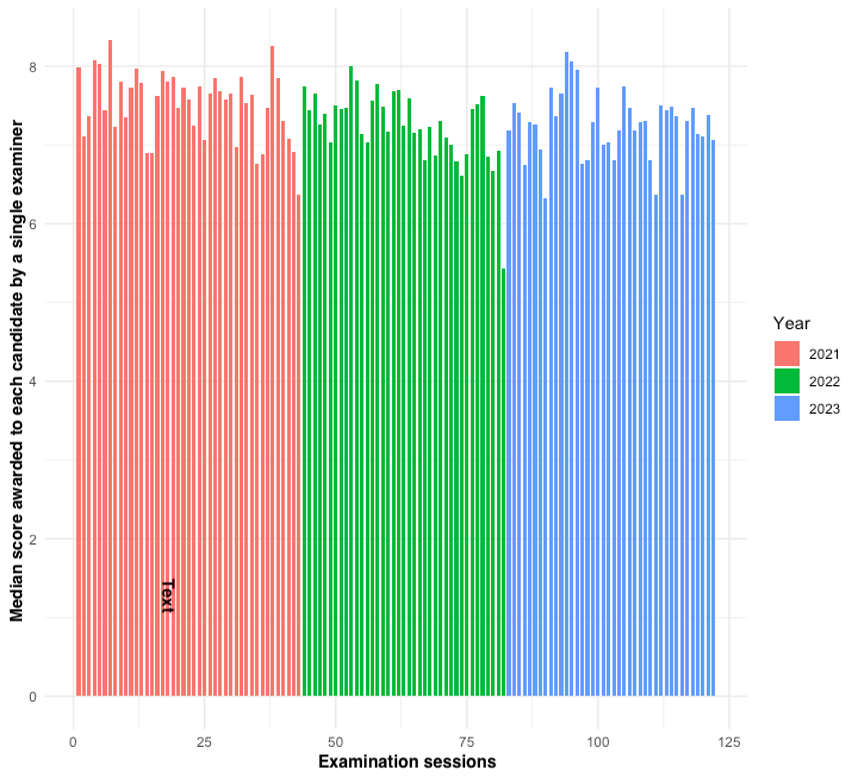

Supplement: Supplemental Digital Content [file ejanet-41-921-s001.jpg]

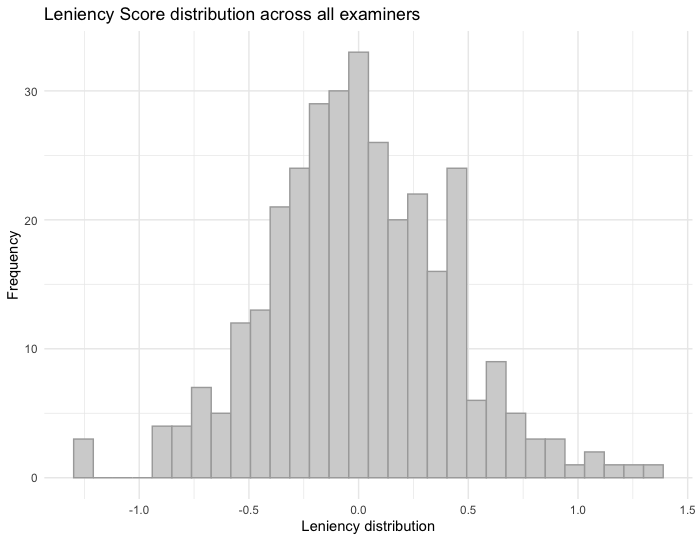


Figure S4: Leniency Score Distribution across all examiners

Supplement: Supplemental Digital Content [file ejanet-41-921-s005.docx]
