# Supplementary material for: Influence of pairing in examiner leniency and stringency (‘hawk-dove effect’) in part II of the European Diploma of Anaesthesiology and Intensive Care: A cohort study
Source: Eur J Anaesthesiol. 2024 Aug 28;41(12):921–31. doi: 10.1097/EJA.0000000000002052 (PMC11556864; doi:10.1097/EJA.0000000000002052)
Supplement: Supplemental Digital Content [file ejanet-41-921-s002.docx]

Figure S2: Distribution of the candidate ability score (θ) as determined by MFRM. This followed a normal distribution curve with some outlier candidates at both extremes. The high standard deviation indicates a wide difference in performance between different candidates.


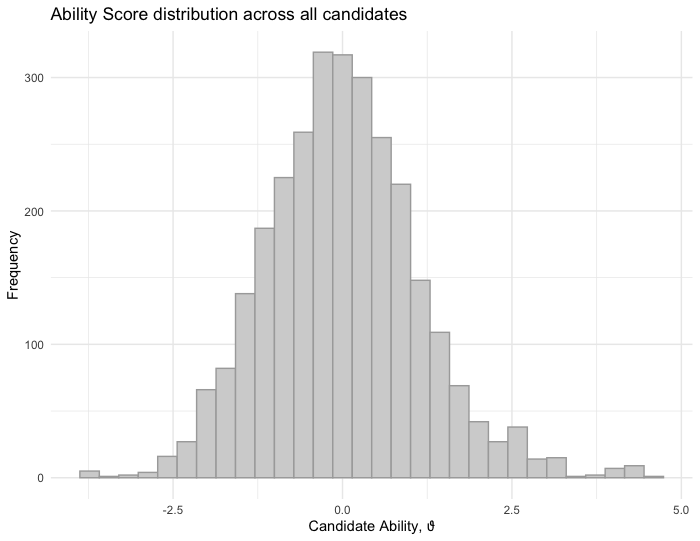


θ
