# Supplementary material for: Influence of pairing in examiner leniency and stringency (‘hawk-dove effect’) in part II of the European Diploma of Anaesthesiology and Intensive Care: A cohort study
Source: Eur J Anaesthesiol. 2024 Aug 28;41(12):921–31. doi: 10.1097/EJA.0000000000002052 (PMC11556864; doi:10.1097/EJA.0000000000002052)
Supplement: Supplemental Digital Content [file ejanet-41-921-s004.docx]

**Figure S4** Average Leniency score among pairs of examiners.


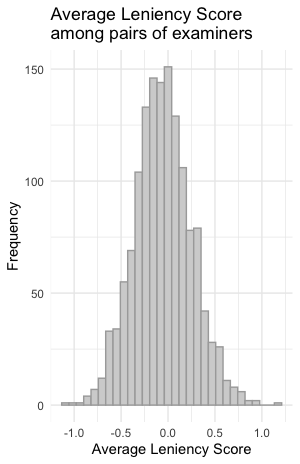


**Figure S3:** Histogram depicting the average Leniency Score among pairs of examiners. The **X-Axis** represents the average leniency score across examiner pairs. The scores range from -1 to 1, where negative values indicate stricter grading than peers, and positive values indicate more lenient grading. A score near 0 suggests a neutral approach.
